# Skeletal Response to Insulin in the Naturally Occurring Type 1 Diabetes Mellitus Mouse Model

**DOI:** 10.1002/jbm4.10483

**Published:** 2021-03-17

**Authors:** Manisha Dixit, Zhongbo Liu, Sher Bahadur Poudel, Gozde Yildirim, Yanjiao Zhang Zhang, Shilpa Mehta, Omer Murik, Geona Altarescu, Yoshifumi Kobayashi, Emi Shimizu, Mitchell B. Schaffler, Shoshana Yakar

**Affiliations:** ^1^ David B. Kriser Dental Center, Department of Molecular Pathobiology New York University College of Dentistry New York New York NY USA; ^2^ Medical Genetics Institute, Shaare Zedek Medical Center Jerusalem Israel; ^3^ Oral Biology Department Rutgers School of Dental Medicine Newark NJ USA; ^4^ Department of Biomedical Engineering City College of New York New York NY USA

**Keywords:** BONE, MICRO‐CT, NOD MICE, OSTEOCYTE, RAMAN SPECTROSCOPY, TYPE 1 DIABETES MELLITUS

## Abstract

Patients with type 1 diabetes mellitus (T1DM) exhibit reduced BMD and significant increases in fracture risk. Changes in BMD are attributed to blunted osteoblast activity and inhibited bone remodeling, but these cannot fully explain the impaired bone integrity in T1DM. The goal of this study was to determine the cellular mechanisms that contribute to impaired bone morphology and composition in T1DM. Nonobese diabetic (NOD) mice were used, along with μCT, histomorphometry, histology, Raman spectroscopy, and RNAseq analyses of several skeletal sites in response to naturally occurring hyperglycemia and insulin treatment. The bone volume in the axial skeleton was found to be severely reduced in diabetic NOD mice and was not completely resolved with insulin treatment. Decreased bone volume in diabetic mice was associated with increased sclerostin expression in osteocytes and attenuation of bone formation indices without changes in bone resorption. In the face of blunted bone remodeling, decreases in the mineral:matrix ratio were found in cortical bones of diabetic mice by Raman microspectroscopy, suggesting that T1DM did not affect the bone mineralization process per se, but rather resulted in microenvironmental alterations that favored mineral loss. Bone transcriptome analysis indicated metabolic shifts in response to T1DM. Dysregulation of genes involved in fatty acid oxidation, transport, and synthesis was found in diabetic NOD mice. Specifically, pyruvate dehydrogenase kinase isoenzyme 4 and glucose transporter 1 levels were increased, whereas phosphorylated‐AKT levels were significantly reduced in diabetic NOD mice. In conclusion, in addition to the blunted bone formation, osteoblasts and osteocytes undergo metabolic shifts in response to T1DM that may alter the microenvironment and contribute to mineral loss from the bone matrix. © 2021 The Authors. *JBMR Plus* published by Wiley Periodicals LLC on behalf of American Society for Bone and Mineral Research.

## Introduction

Type 1 diabetes mellitus (T1DM) is one of the most common chronic pediatric diseases. The incidence of T1DM has been rising globally during the past decades.^(^ ^1^ ^)^ According to the 2014 report The SEARCH for Diabetes in Youth Study: Rationale, Findings, and Future Directions,^(^ ^2^ ^)^ about 200,000 youth (<20 years old) and over a million adults (>20 years old) live with T1DM in the United States. By 2050, it is expected that 600,000 youth and 4 million people over the age of 20 years will live with T1DM in the United States alone. Nearly 20% of patients with T1DM between the ages of 20 and 56 years, meet the criteria for having osteoporosis: reduced bone volume (osteopenia) and BMD.^(^ ^3^, ^4^ ^)^ Furthermore, T1DM associates with complications such as increased risk of fracture^(^ ^5^ ^)^ and impaired bone healing and regeneration.^(^ ^6^ ^)^ Accordingly, a meta‐analysis of approximately 4000 subjects with T1DM showed low BMD at five skeletal sites (whole body, spine, femur, hip, and forearm).^(^ ^1^ ^)^ Currently, treatments of bone disorders in T1DM are similar to those of osteoporosis. Bisphosphonates, which inhibit bone resorption, remain the mainstay of treatment, although the evidence for these therapies in T1DM is lacking.

Although the etiology of skeletal abnormalities in T1DM is multifactorial, uncontrolled glucose levels and the lack of insulin are considered the main causes for T1DM‐associated osteopenia. Insulin is an anabolic hormone, which directly influences bone cells and potentially contributes to the low BMD in T1DM. However, osteoblast differentiation in vitro does not require insulin. Furthermore, global insulin receptor (IR) KO mice that were fully rescued by overexpression of the human IR in pancreas, liver, and brain show normal bone phenotype, suggesting that reduced IR signaling in bone per se is not a major factor contributing to low BMD in T1DM.^(^ ^7^ ^)^ In contrast, a study of mice lacking the IR specifically in osteoblasts show reduced number of osteoblasts on bone surface and decreased trabecular bone volume.^(^ ^8^ ^)^ However, these studies were done during growth, while bone acquisition was peaked and associated with increased body adiposity and impaired glucose homeostasis, both of which indirectly affect bone metabolism. Although the authors claimed that this phenotype stems from the lack of osteocalcin, this is not supported by clinical data from children with T1DM. Systemic markers, including osteocalcin,^(^ ^9^, ^10^ ^)^ RANKL,^(^ ^11^, ^12^ ^)^ leptin,^(^ ^13^, ^14^, ^15^, ^16^ ^)^ PTH,^(^ ^3^, ^17^ ^)^ sclerostin,^(^ ^18^, ^19^, ^20^ ^)^ or vitamin D,^(^ ^21^, ^22^ ^)^ do not show clear relationships with BMD in children with T1DM. In fact, a summary of clinical data from children with T1DM^(^ ^23^ ^)^ supports the notion that the lack of insulin‐like growth factor‐1 (IGF‐1) and hyperglycemia are the main factors contributing to decreased BMD in T1DM. Finally, systemic cytokine levels have been evaluated in a few studies,^(^ ^24^, ^25^, ^26^ ^)^ but their relationships to BMD and fracture have not been established. Thus, the mechanisms leading to T1DM‐associated osteopenia remain to be unraveled.

The autoimmune‐prone nonobese diabetic (NOD) mouse model recapitulates many aspects of human T1DM.^(^ ^27^, ^28^ ^)^ NOD mice show signs of insulitis at approximately 4 to 5 weeks (puberty) and, similar to humans, develop full T1DM at approximately 12 weeks of age (young adult).^(^ ^29^ ^)^ Unlike humans, diabetic NOD (D‐NOD) mice exhibit mild ketoacidosis, allowing them to survive for several weeks after the onset of hyperglycemia without supportive insulin treatment. This allows us to follow the diabetic and insulin‐treated NOD mice simultaneously. Because spontaneous DM develops only in 65% of female and 35% of male NOD mice, our study focused on female mice. Almost every chromosome of the NOD mouse contains at least one gene that affects T1DM development.^(^ ^30^, ^31^ ^)^ The nonobese diabetic‐resistant, NOR strain^(^ ^30^, ^32^, ^33^ ^)^ shares approximately 88% of the NOD genome, but do not develop DM.^(^ ^34^ ^)^ Additionally, because not all NOD mice develop DM, we used NOD mice that did not develop DM (ND‐NOD) as a second control group.

Our goal in the current study was to unravel the cellular and molecular mechanisms that underlay osteopenia in the naturally occurring T1DM NOD mouse model, and their response to insulin treatment. To achieve that goal we studied the morphology, histology, composition, and molecular alterations shortly after DM onset (4 weeks) in NOD mice. Our data indicate early changes in bone matrix mineralization in D‐NOD bones that appear to result from osteoblast/osteocyte activity and relate to their substrate metabolism.

## Materials and Methods

### Animals

Eight‐week‐old female autoimmune‐prone NOD mice (NOD/ShiLtJ, Jax stock#001976) were purchased from Jackson's Laboratory along with *NOR/LtJ* (Jax stock#002050) mice, which are insulitis‐resistant and DM‐free. NOR mice served as controls for morphology studies.

Three batches of mice were studied. Each batch included 20 to 25 NOD and 5 NOR mice. In our facility, only 40% of the female NOD mice developed DM at approximately12 to 14 weeks (contributing to the D‐NOD and the insulin‐treated (In‐NOD) groups). Nondiabetic NOD mice (ND‐NOD) were followed to the time of euthanasia; if they did not develop DM, they were considered ND‐NOD and included in the study. Mice that developed DM after 14 weeks were excluded from the study. Our inclusion criteria were based on an established protocol.^(^ ^35^ ^)^ Briefly, beginning at 10 weeks of age, NOD mice were bled twice weekly via a tail nick, and blood glucose levels were measured. Mice with blood glucose levels of at least 250 mg/dl were retested the following day. Diabetes onset was defined as a nonfasted blood glucose level of at least 250 mg/dl for two consecutive days. Mice that developed DM and were 12 to 14 weeks old were included in the D‐NOD or In‐NOD groups.

ALZET miniosmotic pumps (model 1002) were implanted subcutaneously in all the mice. NOR mice were given ALZET pumps filled with sterile diluent at 12 to 14 weeks of age and sacrificed at 16 to 19 weeks of age. D‐NOD mice were given ALZET pumps filled with diluent 2 days after detection of DM, and included in the study only if DM occurred between 12 and 14 weeks of age. NOD mice implanted with diluent‐filled pumps that did not develop DM by 12 to 14 weeks were included in the ND‐NOD group, only if they remained nondiabetic until the end of the study. Insulin treatment was given via ALZET pumps filled with Humulin R diluted to 41.67 U/mL (corresponding to a dose of 0.25 U daily) using sterile diluent on the third day after detection of DM, and only if they were 12 to 14 weeks old. Mice were euthanized 4 weeks after pump implantation.

Mice were monitored twice a week for nonfasted blood glucose levels. Hypoglycemia was defined as nonfasted blood glucose levels lower than 30 to 40 mg/dl Mice exhibiting a blood glucose level of <40 mg/dl with or without clinical signs of poor DM management (more than 10% body weight loss, lethargy, rough hair coat) were provided lactated Ringers solution with 5% dextrose (1 mL/mouse) once daily via s.c. injection, as needed. Mice with blood glucose >25 mg/dl with clinical signs of poor DM were removed from the study. Mice were followed for 4 weeks following s.c. insertion of an osmotic pump. Tissue dissection of either diabetic or insulin‐treated NOD mice always included age‐matched ND‐NOD and NOR mice.

### Micro‐computed tomography

μCT was performed according to published guidelines.^(^ ^36^ ^)^ We used a high‐resolution SkyScan μCT system (SkyScan 1172; Bruker). Images were acquired using a 10‐MP digital detector, 10‐W energy (70 kV and 142 mA), and a 0.5‐mm aluminum filter with a 9.7‐μm image voxel size for femurs and vertebra, 7.5 μm for the mandible. A fixed global threshold method was used based on the manufacturer's recommendations and preliminary studies, which showed that mineral variation between groups was not high enough to warrant adaptive thresholds. The cortical region of interest (ROI) in the femur was selected as the 2.0‐mm mid‐diaphyseal region directly below the third trochanter, which includes the mid‐diaphysis and more proximal cortical regions. The trabecular measurements were taken at the femur distal metaphysis 2.5 mm below the growth plate. The L5 vertebra was used to assess the trabecular bone morphology of the axial skeleton. The volume of interest (VOI) of alveolar bone in the mandible was chosen as 80 slices between roots of the first molar, such that a 600‐um vertical length was selected. Following VOI selection, a ROI of an elliptical shape was selected and applied to all samples. Data were analyzed using CTAn software (version 1.17.7.2+; Bruker μCT).

### Histomorphometry and histology

Mice were injected with calcein (10 mg/kg) at day 10 and day 2 before euthanasia. Femors were dissected, fixed in 10% neutral buffered formalin, dehydrated, and embedded undecalcified in methyl methacrylate (MMA). Longitudinal sections of the distal femurs and the mid diaphysis were used to measure parameters of bone formation using Osteo v18.2.60 (BIOQUANT Image Analysis Corp). Mineralizing surface per bone surface (MS/BS; %) and mineral apposition rate (MAR; μm/d) were measured in unstained 10‐ to 20‐μm sections under fluorescent light. The bone formation rate per bone surface (BFR; μm^3^/μm^2^/d) was calculated. The terminology and units used are those recommended by the Histomorphometry Nomenclature Committee of the American Society for Bone and Mineral Research.^(^ ^37^ ^)^

For immunostaining, femurs were fixed in 10% zinc formalin and were decalcified using EDTA. Bones were then processed for paraffin embedding and sectioning. Sections (7 m) were stained with tartrate‐resistant acid phosphatase (TRAP; Sigma, 387A‐1KT), Cathepsin K, Glut1, PDK4, and p‐AKT antibodies (Supplementary Information Table S1).

### Raman microspectroscopy

Raman microspectroscopy (RS) was performed according to Morris et al.^(^ ^38^ ^)^ RS was acquired using a Thermo Fisher Scientific DXR2 confocal Raman microscope, with 785‐nm wavelength excitation at 50× magnification objective. Raman data were collected from the posterior quadrant of PMMA embedded femur mid‐diaphysis. Our principal focus was on: (i) mineral:matrix ratio (ν1PO_4_ [950–970] peak:amide I [1660–1690] collagen peak), (ii) carbonate:phosphate ratio (1050–1070 and the 950–970 peaks, respectively), and (iii) mineral crystallinity (full width at half max of the PO_4_ peak). Analyses were performed according to Morris et al^(^ ^38^ ^)^ as in our recent studies.

### Gene expression

Total RNA was extracted using TRIzol (Invitrogen) or by RNeasy Plus kit (Catalog no. 74134; QIAGEN), reverse‐transcribed to cDNA (Catalog no. 18080‐051; Life Technologies), and subjected to real‐time PCR using SYBR master mix (Catalog no. 4385612; Life Technologies/Applied Biosystems) on a BioRad CFX384 real‐time machine. Transcript levels were assayed three times in each sample, and corrected to 18S. The primer sequences are detailed in Supplementary Information Table S2.

### RNAseq

RNA was isolated from alveolar bone of the mandible (rich in osteocytes) of D‐NOD (n = 3 pools/group; each pool contains RNA extracts from three mice) insulin‐treated, and aged matched ND‐NOD mice (similar design). RNAseq was performed by GENEWIZ and included ribosomal RNA depletion.

After extraction of gene hit counts, the gene hit counts table was used for downstream differential expression analysis. Using DESeq2, a comparison of gene expression between the groups was performed. The Wald test was used to generate *p* values and log2‐fold changes. Genes with an adjusted *p* value <0.05 and absolute log2 fold change >1 were called as differentially expressed genes for each comparison. A gene‐ontology analysis was performed on the statistically significant set of genes by implementing the software GeneSCF v.1.1‐p2. The mgi GO list was used to cluster the set of genes based on their biological processes and to determine their statistical significance. A list of genes clustered based on their gene ontologies was generated.

### Statistical analysis

The numbers of animals used were calculated based on previous experience and N = 4*Zα*(S^2^/W^2^), where N = sample size, Zα = 1.96 for a = 0.05 or 1.645 for a = 0.01, S = standard deviation of the variable, and W = desired total width of the confidence interval (CI). Accordingly, 8 to 10 mice were needed for compositional and morphological studies, 6 to 8 mice for histomorphometry, and 5 to 6 mice for immunohistochemistry studies, to satisfy a 90% CI. Data are presented as means ± SEM. Differences between groups were tested using one‐way ANOVA and post hoc Tukey's test as appropriate. Significance accepted at *p* < 0.05. Researchers were blinded to sample identification in all experimental procedures.

## Results

### The NOD experimental model

**Fig. 1:**
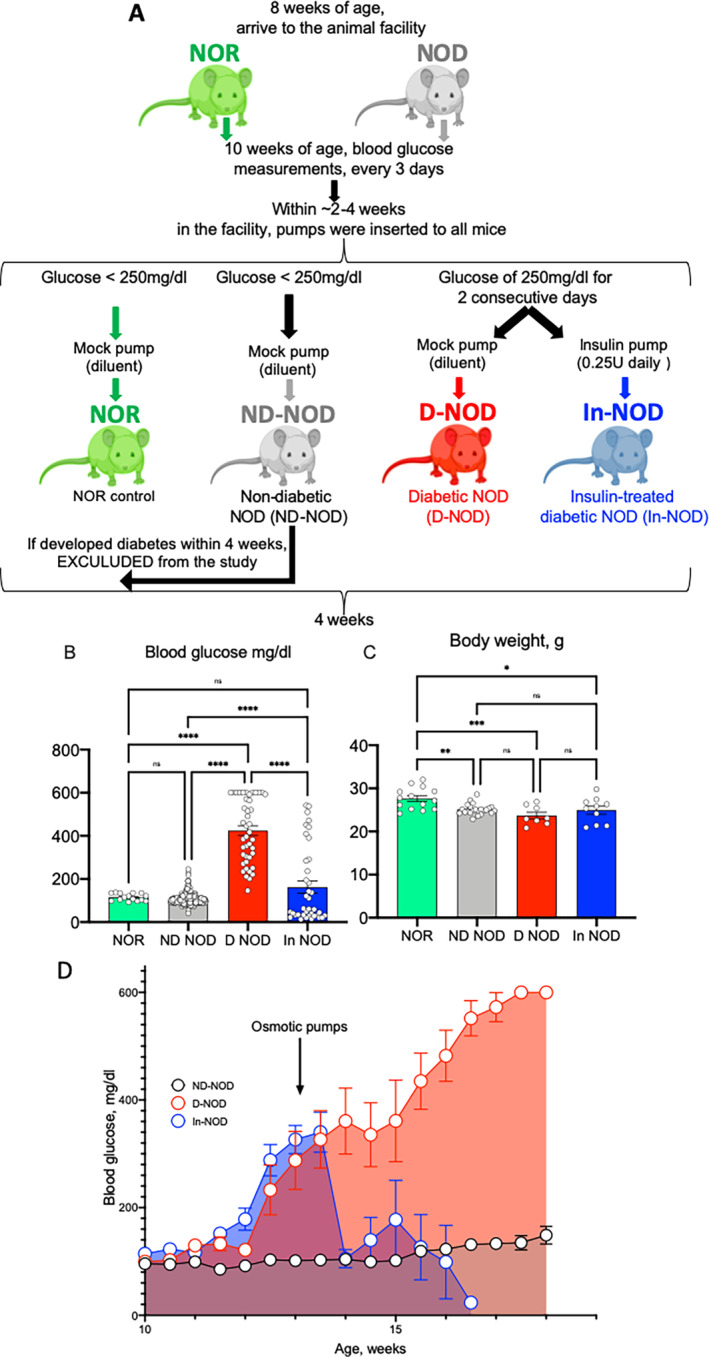
Nonobese diabetic (NOD) mice exhibit fourfold elevations in blood glucose and decreased body weight. (*A*) Schematic presentation of the experimental design. (*B*) Blood glucose levels measured throughout the study. (*C*) Body weight recorded at the end of the study. (*D*) Longitudinal measurements of blood glucose. Values are given as mean ± SEM; and *p < 0.05 considered significant (nonobese diabetic‐resistant [NOR], n = 14; nondiabetic NOD mice [ND‐NOD], n = 19; diabetic NOD [D‐NOD], n = 8, insulin‐treated [In‐NOD], n = 10).

Our study design (Fig. 1*A*) included the use of female NOD mice and the NOR mice,^(^ ^30^, ^34^ ^)^ along with age‐matched mice (that did not develop DM until the time of tissue collection, as per our criteria detailed in the Materials and Methods section). Insulin treatment was given via osmotic pumps. Controls and diabetic mice were given a pump with diluent only. D‐NOD mice developed at approximately 12 to 14 weeks of age, with blood glucose of 405 ± 23 mg/dl (Fig. 1*B*,*D*). Four weeks of insulin supplementation reduced blood glucose to 147 ± 26 (although several hyper‐ and hypoglycemic events were recorded). Over a course of 4 weeks, hyperglycemia caused significant reduction in body weight (Fig. 1*C*), which was attributed to a loss of adipose tissue and muscle mass (data not shown). Insulin treatment restored body weight to control levels. Mice were euthanized at approximately 16 to 19 weeks of age.

### T1DM in NOD mice impaired skeletal morphology

**Fig. 2:**
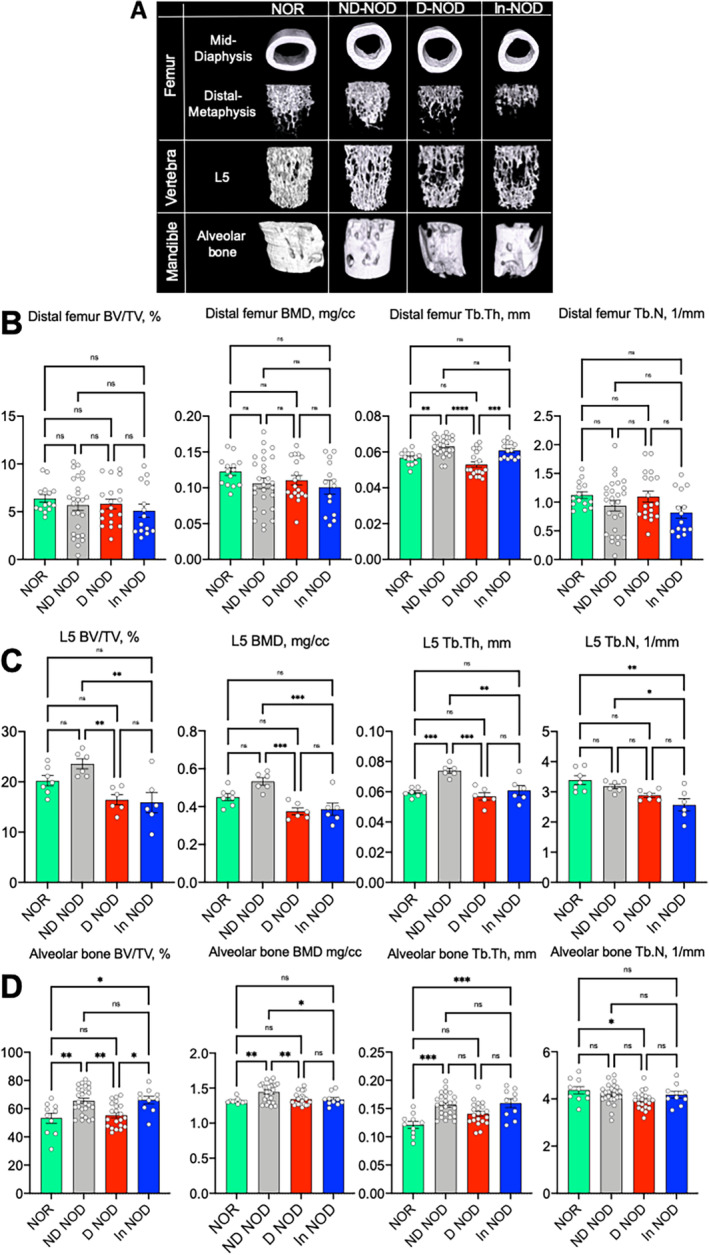
Insulin treatment of diabetic NOD mice has site‐specific effects on skeletal parameters. (*A*) Three‐dimensional images of μCT imaging (9.7 μm) taken at the femur mid‐diaphysis, femur distal metaphysis, L5 vertebra, and the mandible. (*B*) Trabecular bone parameters (bone volume/total volume [BV/TV], trabecular BMD, trabecular thickness [Tb.Th], and trabecular number [Tb.N]) taken at the femur distal metaphysis. (*C*) Cancellous bone parameters of the L5 vertebral body. (*D*) Alveolar bone parameters taken in the mandible. Each dot on the bar graph represents one sample. Values are given as mean ± SEM; *p < 0.05 was considered significant. Sample size for each site and group is indicated in Supplementary Information Table S3. D‐NOD, diabetic NOD; In‐NOD, insulin‐treated NOD; ND‐NOD, nondiabetic NOD; NOD, nonobese diabetic; NOR, nonobese diabetic‐resistant

Three sites of the appendicular and axial skeleton were analyzed by μCT. We found that skeletal response to DM and insulin treatment in this naturally occurring T1DM model was heterogeneous (Fig. 2, Supplementary Information Table S3). Cortical bone of the appendicular skeleton, analyzed at the femur mid‐diaphysis, showed minor decreases in total cross‐sectional area (T.Ar) in ND‐NOD mice as compared with the NOR mice and in D‐NOD mice as compared with ND‐NOD mice. These changes were not accompanied by increases in marrow area (M.Ar), indicating an inhibition of periosteal expansion. Minor changes were also observed in bone area (B.Ar) and cortical bone thickness (Cs.Th) in D‐NOD mice that were mostly recovered by insulin treatment (Supplementary Information Table S3, Fig. 2*A*). Surprisingly, the metabolically active, cancellous bone at the distal metaphysis of the femur did not show significant changes in bone volume/total volume (BV/TV) and BMD. A significant decrease (25%) in trabecular thickness (Tb.Th) was found in D‐NOD mice as compared with ND‐NOD mice that was recovered by insulin treatment (Supplementary Information Table S3, Fig. 2*A*,*B*). More obvious differences were detected in the cancellous bone of the L5 vertebra. We found significant reductions in BV/TV (40%), BMD (30%), and Tb.Th (25%) in D‐NOD as compared with ND‐NOD mice that were not recovered with insulin treatment (Supplementary Information Table S3, Fig. 2*A*,*C*). Finally, alveolar bone at two anatomical sites in the mandible showed reductions in BV/TV in diabetic mice, which was recovered by insulin. However, despite recovery of BV/TV with insulin, the decreased BMD (10%) of alveolar bone was not restored to the ND‐NOD levels (Supplementary Information Table S3, Fig. 2*A*,*D*). It is important to note that we did not detect major changes in bone morphology, likely because it was assessed shortly after DM onset (4 weeks). Additionally, many of the bone traits significantly differed between NOR and the ND‐NOD mice, suggesting that bone changes in the ND‐NOD mice may have preceded the full‐blown diabetic stage seen in the D‐NOD (representing in fact prediabetic changes in bone morphology). Finally, we should note that insulin treatment did not fully recover the morphological changes in bone likely because of insufficient blood glucose control as evidenced by episodes of hypo‐ and hyperglycemia (Fig. 1*B*).

### T1DM in NOD mice associated with reduced‐expression bone‐cell–lineage markers

**Fig. 3:**
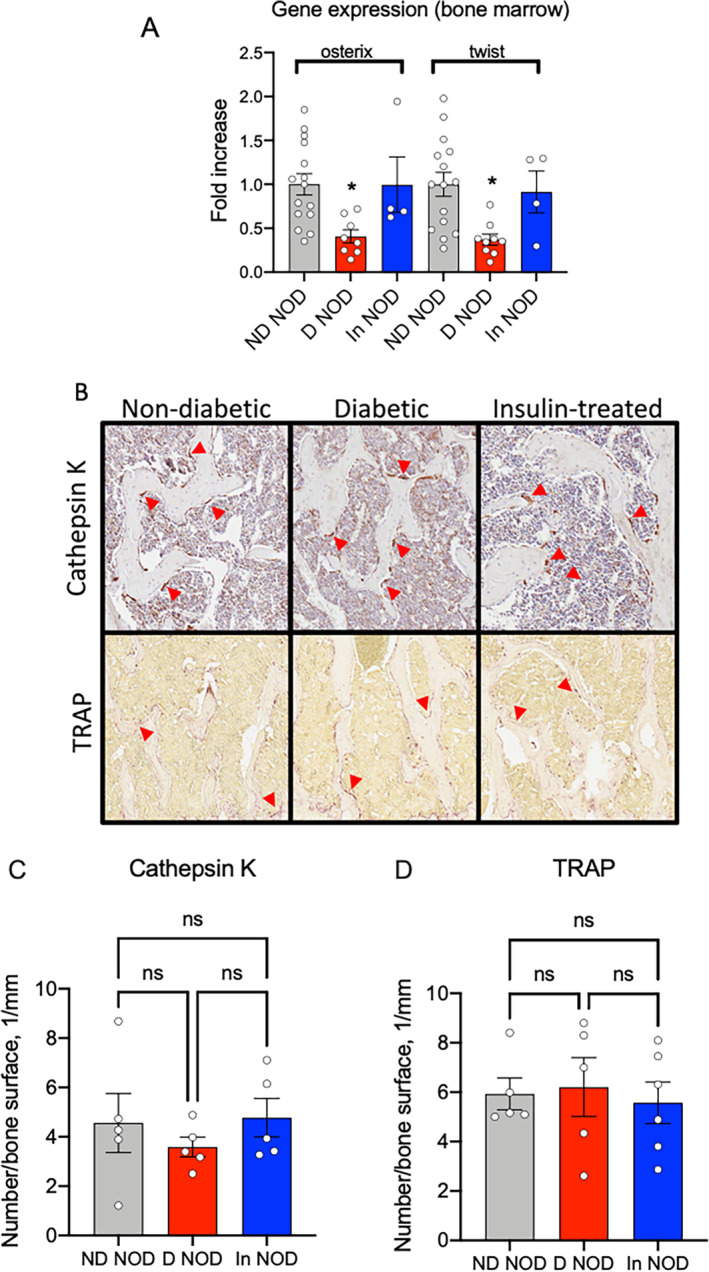
Diabetic NOD mice show reduced bone marrow osteoprogenitor markers without differences in osteoclast number on bone surface. (*A*) Expression of *osterix* and *twist* was evaluated in total RNA extracted from bone marrow of the femur and tibia. (*B*) Cathepsin K‐positive and TRAP‐positive cells on bone surface were visualized and quantified (*C*,*D*) by immunohistochemistry of the L5 vertebra. Each dot on the bar graph represents one sample. Values are given as mean ± SEM; *p < 0.05 was considered significant. D‐NOD, diabetic NOD; In‐NOD, insulin‐treated NOD; ND‐NOD, nondiabetic NOD; NOD, nonobese diabetic; NOR, nonobese diabetic‐resistant; ns, nonsignificant; TRAP, tartrate‐resistant acid phosphatase

Characterization of osteoprogenitor markers in the marrow of long bones showed significant reductions in *osterix* and *twist* in D‐NOD mice that were recovered with insulin (Fig. 3*A*), which suggests that factors other than osteoprogenitor number play a role in the incomplete bone recovery of the insulin‐treated diabetic NOD mice. Markers of osteoblasts and osteocytes in cortical bone of femur or tibia, as well as alveolar bone of the mandible, varied between the sites but showed a tendency to reduce in D‐NOD mice and were mostly recovered with insulin treatment (Supplementary Information Fig. S1).

Expression of osteoclast markers did not differ between ND‐NOD, D‐NOD, or In‐NOD mice (Supplementary Information Fig. S2). Further, the number of cathepsin K or TRAP‐positive (osteoclast) cells on cancellous bone surfaces of L5 vertebra did not differ between the groups (Fig. 3*B*‐*D*). Osteoclast numbers in other skeletal sites were not studied and may suggest otherwise. However, our observations are in accordance with other studies indicating that bone resorption is not the driver of DM‐associated osteopenia.

### Osteocytes of T1DM‐NOD showed increased sclerostin expression and exhibited impaired bone remodeling

**Fig. 4:**
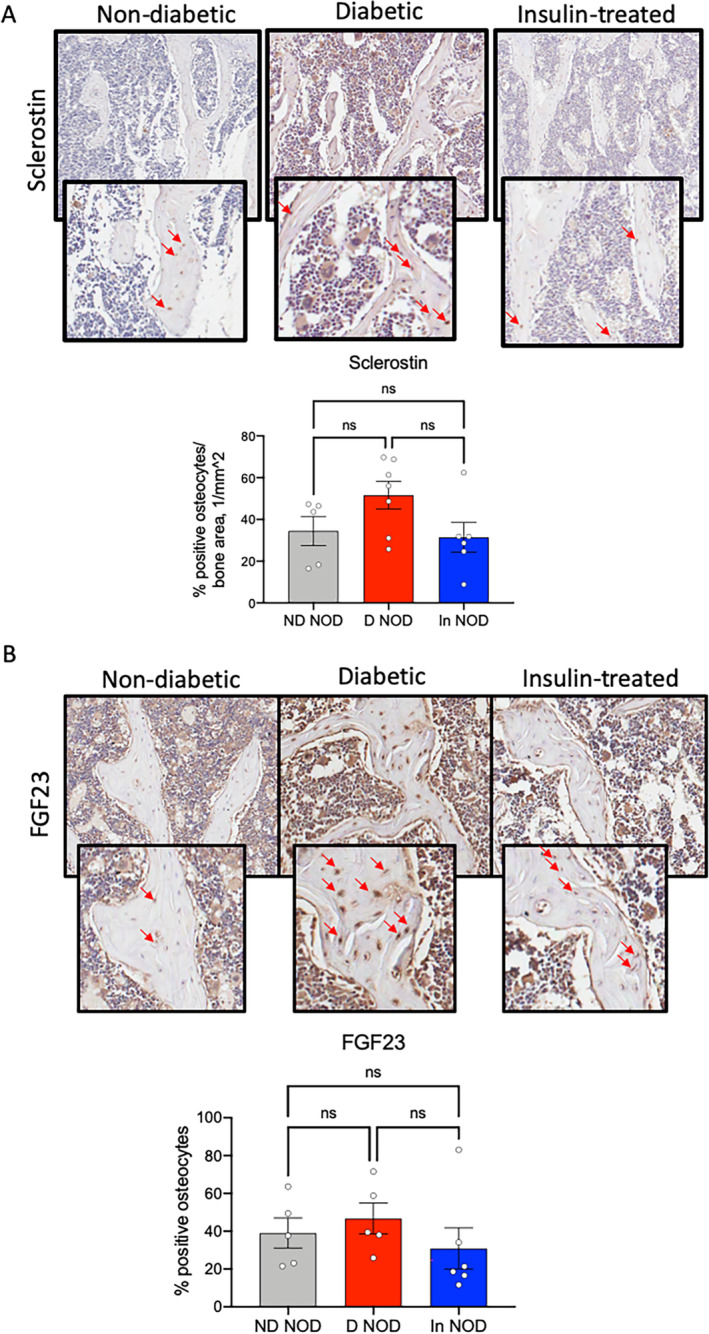
Diabetic NOD mice show increased number of sclerostin and FGF23‐positive osteocytes. Sclerostin‐ (*A*) and FGF23‐ (*B*) positive osteocytes in cancellous bone of the L5 vertebra were visualized and quantified by immunohistochemistry. Each dot on the bar graph represents one sample. Values are given as mean ± SEM; *p < 0.05 was considered significant. D‐NOD, diabetic NOD; In‐NOD, insulin‐treated NOD; ND‐NOD, nondiabetic NOD; NOD, nonobese diabetic; NOR, nonobese diabetic‐resistant; ns, nonsignificant

Because trabecular bone volume in the femur distal metaphysis was significantly reduced in D‐NOD mice, we performed our in situ immunohistochemistry studies in the trabecular bone of the L5 vertebra. This site provided a larger bone surface area to assess cellular abnormalities in response to T1DM. Several studies have indicated increases sclerostin, an endogenous inhibitor of the anabolic Wnt1 pathway, in response to T1M. We found that the D‐NOD group showed increases in overall percentage of sclerostin positive cells in cancellous bone of the L5 vertebra (Fig. 4*A*), but because of large variability, this did not reach significance. The levels of FGF23, an osteocyte‐derived phosphaturic hormone that is inhibited in response to insulin,^(^ ^39^ ^)^ were elevated in D‐NOD and reduced in response to insulin in the In‐NOD mice (Fig. 4*B*), but because of large variability, this did not reach significance.

**Fig. 5:**
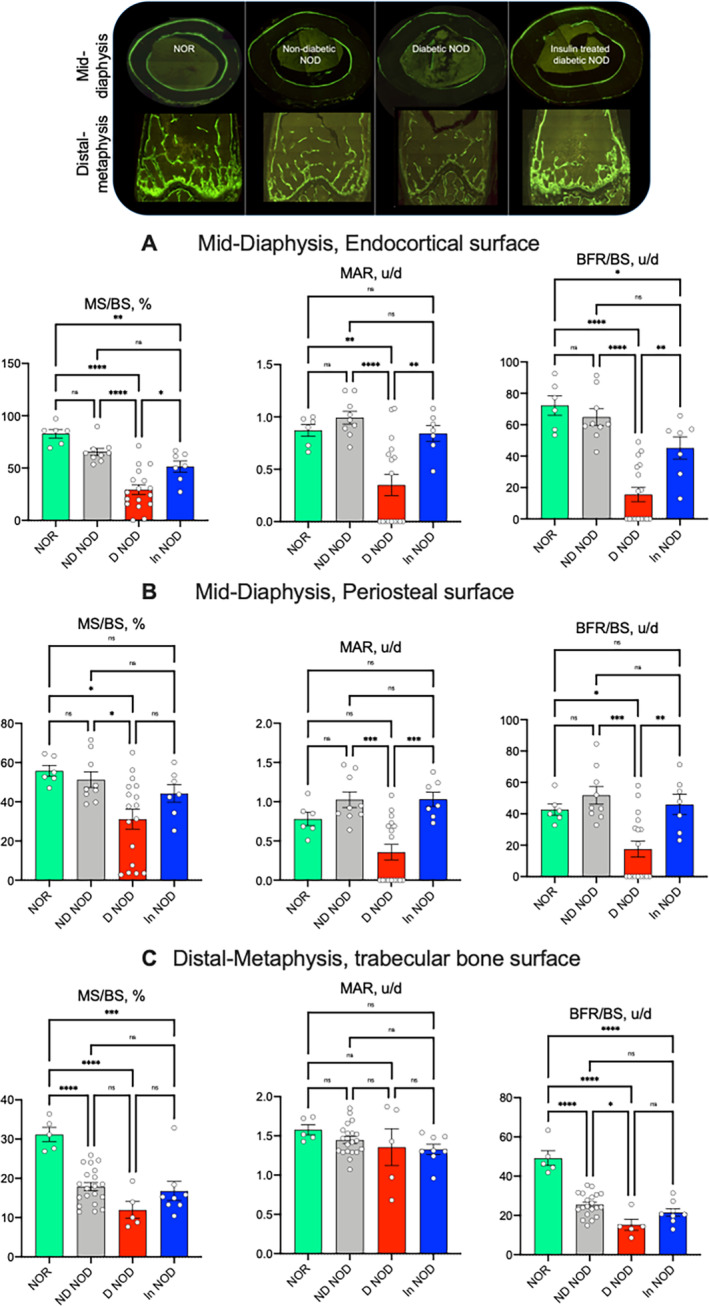
Diabetic NOD mice exhibit inhibited bone formation. Dynamic bone indices were determined by histomorphometry of bones from mice injected with calcein at 7 days distance. Cortical bone formation indices were taken at the endocortical surface (*A*) and the periosteal surface (*B*) of the femur mid‐diaphysis included mineral surface to bone surface (MS/BS) ratio, mineral apposition rate (MAR), and bone formation rate (BFR). (*C*) Similar indices were taken of the trabecular bone compartment at the femur distal metaphysis. Each dot on the bar graph represents one sample. Values are given as mean ± SEM; *p < 0.05 was considered significant. D‐NOD, diabetic NOD; In‐NOD, insulin‐treated NOD; ND‐NOD, nondiabetic NOD; NOD, nonobese diabetic; NOR, nonobese diabetic‐resistant

Next, we studied dynamic indices of bone remodeling in the femur by histomorphometry. We found significant reductions in the percentage of mineralized surface to bone surface (MS/BS) ratio, significant reductions in mineral apposition rate (MAR), and bone formation rate (BFR) at the endosteal surfaces of the femur mid‐diaphysis in D‐NOD mice (Fig. 5*A*). The MS/BS ratio, MAR, and BFR in the endocortical surface of NOR mice did not differ significantly from that of ND‐NOD mice. Interestingly, insulin treatment (In‐NOD) did not fully recover MS/BS in the endosteal surface (Fig. 5*A*), whereas all parameters were recovered in the periosteal surface (Fig. 5*B*). Analyses of trabecular bone at the femur distal metaphysis (Fig. 5*C*) showed reduced MS/BS and BFR in D‐NOD mice, as well as in In‐NOD mice as compared with the NOR mice. All together these data suggest an impaired bone remodeling in D‐NOD mice.

### T1DM in NOD mice resulted in differentially regulated genes involved in matrix composition and cellular metabolism in bone

**Fig. 6:**
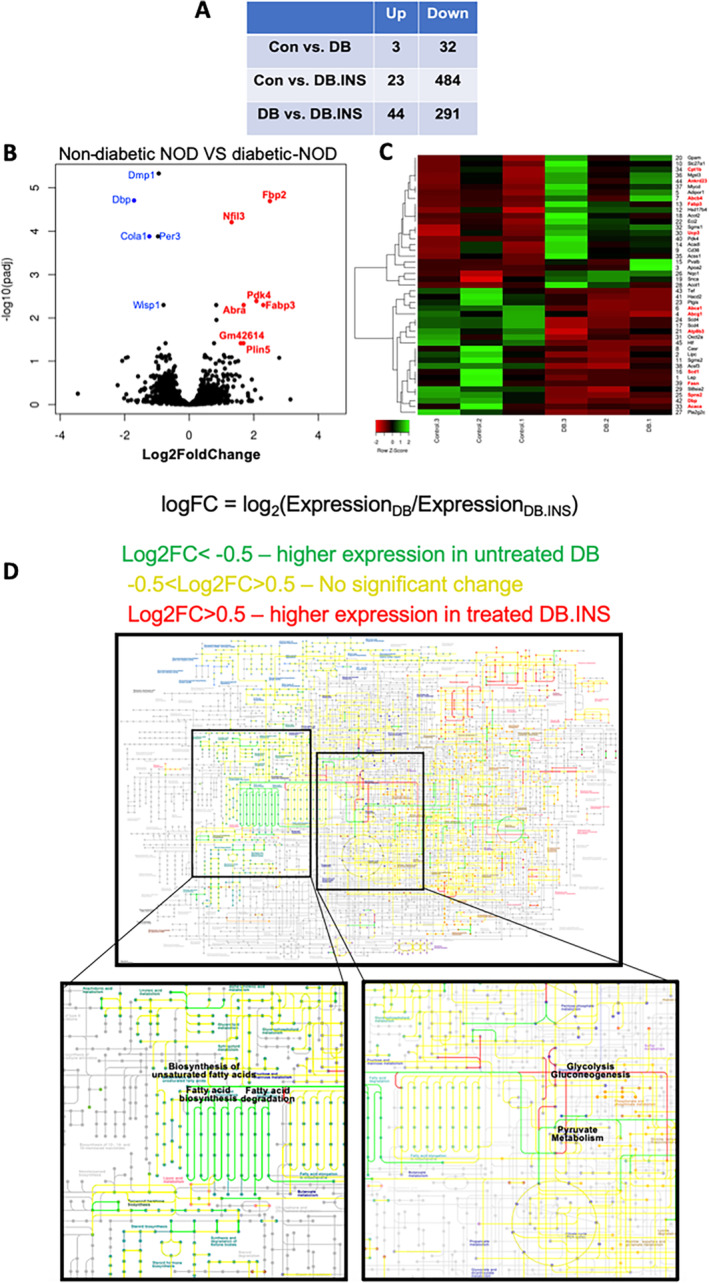
RNAseq data from alveolar bone showing differentially regulated genes involved in matrix composition and cellular metabolism in bone. Whole transcriptome sequencing was performed using RNA extracts depleted of ribosomal RNA. Hit counts were compared between the groups and subjected to gene ontology analyses. The Wald test was used to generate *p* values and log2‐fold changes. Genes with an adjusted *p* value <0.05 and absolute log2‐fold change >1 were called differentially expressed genes for each comparison. (*A*) A table summarizing genes that were up‐ and downregulated in each group. (*B*) Volcano plot showing significantly differentially expressed genes between nondiabetic and diabetic NOD mice. (*C*) Hit map of genes involved in lipid metabolism, a comparison between nondiabetic and diabetic NOD mice. (*D*) Significantly differentially expressed genes imported to the KEGG database maps. We found an impaired expression of genes involved in lipid metabolism (labeled in green). Specifically, these genes were elevated in diabetic NOD mice as compared with insulin‐treated diabetic NOD. DB, diabetes; CON, control; INS, insulin; NOD, nonobese diabetic

To expand our understanding of the effects of T1DM on bone cell metabolism we performed transcriptome (RNAseq) studies. Comparisons between ND‐NOD (group A, control), diabetic NOD (DB, group B), and insulin‐treated NOD (DB‐ins, group C) mice revealed several differentially regulated genes (Fig. 6*A*). Significantly differentially expressed genes are presented in a volcano plot (Fig. 6*B*). As expected, among the downregulated genes, *Col1a* (blue) was significantly reduced in diabetic as compared with the ND‐NOD mice. Interestingly, among the upregulated genes in diabetic mice was pyruvate dehydrogenase kinase isoenzyme 4 (PDK4; red), which is a mitochondrial enzyme that regulates glucose oxidation.^(^ ^40^ ^)^ Consequently, genes were clustered by their gene ontology, and the enrichment of gene ontology terms was tested using Fisher's exact test (GeneSCF v1.1‐p2). In accordance with upregulation of PDK4, which predicted a shift in substrate metabolism, a heat map (Fig. 6*C*) and enriched KEGG pathway analyses (Fig. 6*D*) showed dysregulation of genes involved in fatty acid oxidation, transport, and synthesis in the D‐NOD mice. KEGG pathway analysis revealed that insulin‐treated NOD mice upregulated genes involved in glycolysis and pyruvate metabolism.

**Fig. 7:**
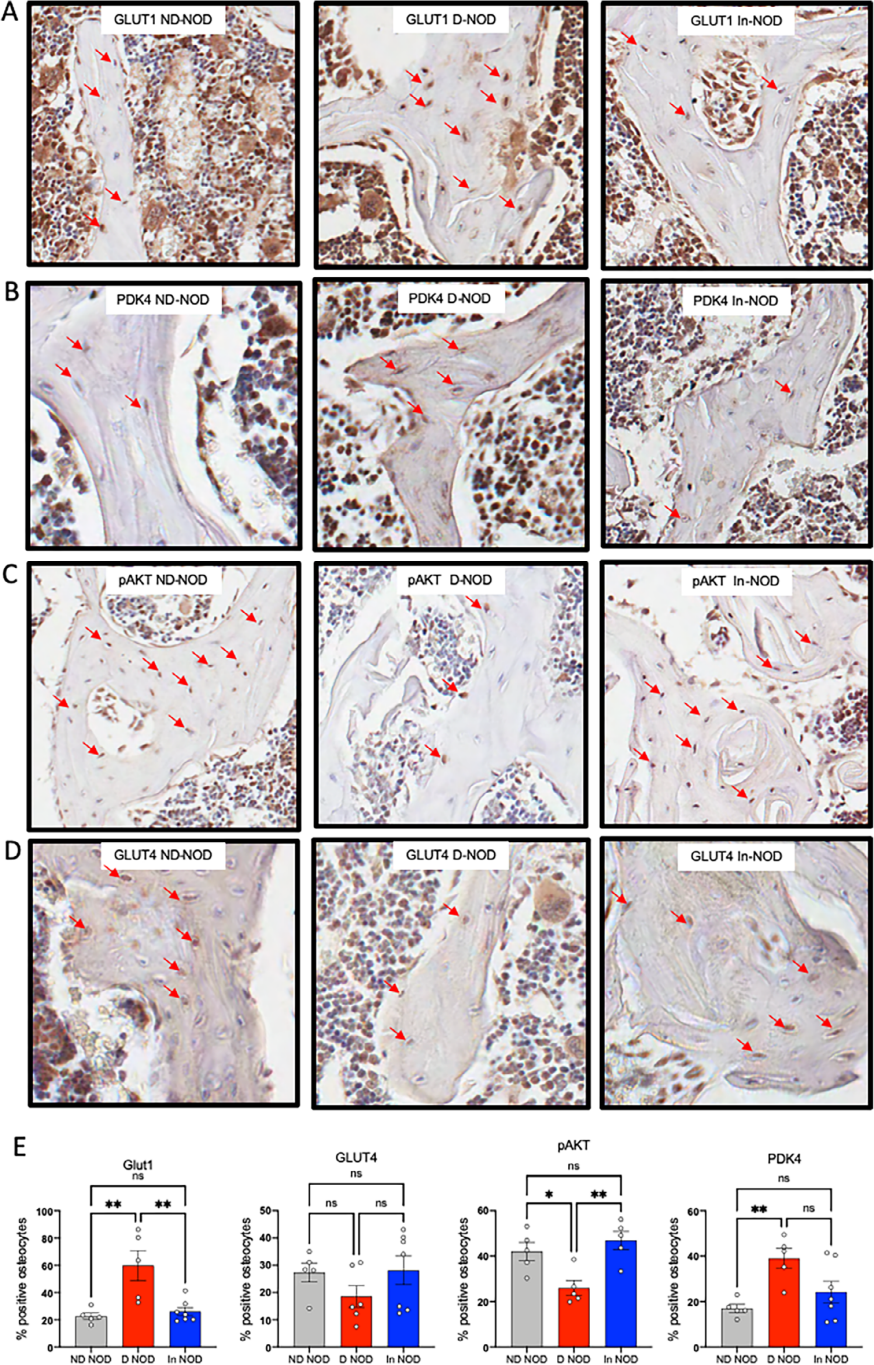
Diabetic NOD mice show alterations in the expression of GLUT1, PDK4, and p‐AKT. GLUT1 (*A*), PDK4 (*B*), (*C*) pAKT, and (*D*) GLUT4‐positive osteocytes in cancellous bone of the L5 vertebra were visualized and quantified (*E*) by immunohistochemistry. Each dot on the bar graph represents one sample. Values are given as mean ± SEM; *p < 0.05 was considered significant. D‐NOD, diabetic NOD; In‐NOD, insulin‐treated NOD; ND‐NOD, nondiabetic NOD; NOD, nonobese diabetic; NOR, nonobese diabetic‐resistant

GLUT1 is the predominant glucose transporter in osteoblasts cells and likely in osteocytes. In view of the elevated glucose levels, we studied the levels of this transporter in osteocytes of trabecular bone in the L5 vertebra. We found increases in overall percentage of GLUT1‐positive cells, as well as in the number of GLUT1‐positive osteocytes (Fig. 7 *A*, Supplementary Information Fig. S2). In agreement with our transcriptome studies, we also found upregulation in PDK4 protein levels in D‐NOD mice by immunohistochemistry (Fig. 7 *B*, Supplementary Information Fig. S2). On the other hand, phosphorylated AKT (p‐AKT) levels were reduced in D‐NOD mice and increased in response to insulin treatment (Fig. 7*C*, Supplementary Information Fig. S2). Insulin‐dependent glucose uptake occurs via GLUT4. We found that GLUT4 levels decreased in the D‐NOD mice and normalized with insulin treatment (Fig. 7*D*, Supplementary Information Fig. S2); however, because of in‐group variability, it did not reach significance.

### T1DM in NOD mice altered bone matrix composition

**Fig. 8:**
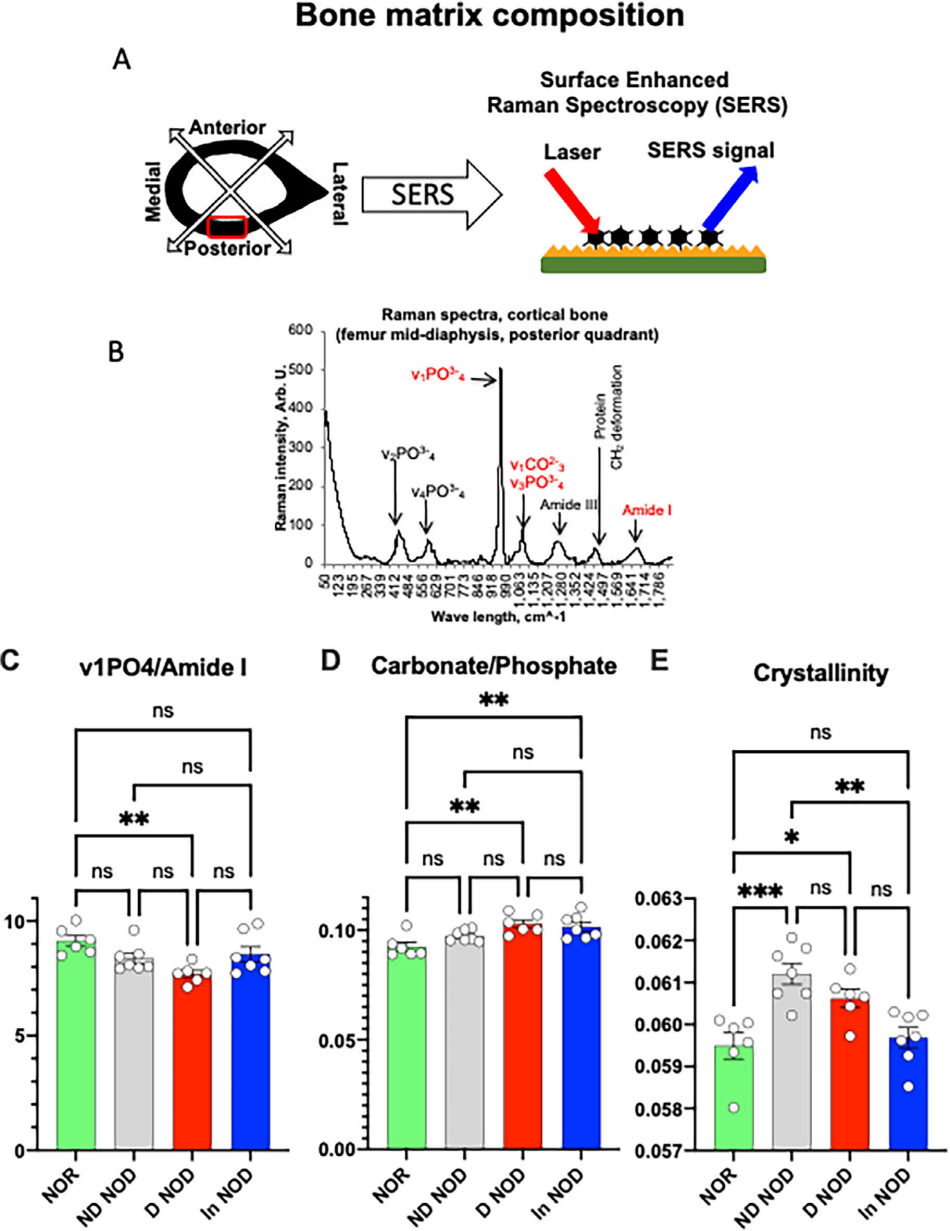
Diabetic NOD mice show compromised bone matrix composition. (*A*) Schematic presentation of the cortical quadrant site analyzed by surface‐enhanced Raman spectroscopy. (*B*) A typical Raman spectrum of a mouse cortical bone. Peaks are labeled according to Morris et al.^(^ ^38^ ^)^ (*C*) Mineral:matrix ratio, (*D*) carbonate:phosphate ratio, and (*D*) mineral crystallinity. Each dot on the bar graph represents a mean of 15 to 20 spectra per mouse taken at the posterior quadrant of the femur. Values are given as mean ± SEM; *p < 0.05 was considered significant. D‐NOD, diabetic NOD; In‐NOD, insulin‐treated NOD; ND‐NOD, nondiabetic NOD; NOD, nonobese diabetic; NOR, nonobese diabetic‐resistant; ns, nonsignificant

RS was performed on cortical cross sections from femurs (Fig. 8*A*) and spectra analyzed according to Morris et al^(^ ^38^ ^)^ (Fig. 8*B*). We found reduced mineral/matrix ratio in the D‐NOD mice that was increased with insulin treatment (Fig. 8*C*). The carbonate:phosphate ratio was significantly increased in D‐NOD and In‐NOD mice as compared with the NOR controls (Fig. 8*D*). Crystallinity is a metric related to mineral size. Mineral crystallinity was significantly increased in D‐NOD and ND‐NOD mice as compared with NOR control mice (Fig. 8*E*). Notably, as seen in our D‐NOD mice, in osteoporotic bone, mineral content is reduced, but the mineral crystal size is increased and the carbonate:phosphate ratio is increased.^(^ ^41^ ^)^ An increase in mineral particle size is also seen in aged bone and is thought to contribute to increased bone fragility during aging.^(^ ^42^ ^)^

## Discussion

T1DM in NOD mice resulted in changes in bone structural morphology, reduced BMD, and bone mineral:matrix ratio. Reductions in bone mineral content occurred in face of blunted bone formation with no detected differences in bone resorption, suggesting microenvironmental changes that favor mineral loss. Our transcriptome data, partially supported by histology, indicate changes in the expression of genes involved in glucose and lipid metabolism in bones of D‐NOD mice.

Numerous studies have utilized the streptozotocin (STZ)‐induced β‐cell death to model T1DM in mice. Although informative, the STZ model does not recapitulate well human T1DM and has limitations, including an intact immune system, acute (and almost immediate) reductions in β‐cell mass, and skeletal toxicity from STZ. We chose to use the NOD mouse model to study the effects of T1DM on the skeleton. This model mimics well the compromised immune system in humans and the gradual insulitis leading to DM. However, the NOD model also has limitations. These include development of DM only in a fraction of the mice (in our case 40% of the NOD females developed T1DM), and varied ages of onset of DM (from 12–30 weeks of age). To overcome these caveats, we used three cohorts of mice (to achieve sufficient sample size) and included two control groups: the NOR mice that were DM resistant and age‐matched NOD mice that did not develop DM as per our criteria.

To better interpret our data, it is important to put forward a few limitations inherent in the NOD model. As seen in the progression of the human disease, the NOD mice display a wide blood glucose excursion (100‐300 mg/dl) at the prediabetic stage. Based on a single daily measurement of blood glucose levels, it was hard to determine whether the ND‐NOD mice were truly DM‐free. We believe that the reason we did not detect significant differences between the D‐NOD and the ND‐NOD mice in a few of the tested traits is, in part, because we were not able to differentiate between the prediabetic and the “truly” ND mice. Nonetheless, the included group of ND‐NOD mice indicates that some of the bone impairments start at the prediabetic stages and get worse with the development of DM. The possibility that bone changes could precede the insulin intervention may also explain the failure to fully rescue bone mass in the In‐NOD mice. Additionally, we note that, similar to the human T1DM, insulin intervention does not provide tightly controlled blood glucose levels. As seen in Fig. 1*B*, the In‐NOD group experienced hyper‐ and hypoglycemic events that partially explain the failure of insulin to fully rescue the bone phenotype. Despite the aforementioned limitations, we bring new insights into the mechanism underlying T1DM‐associated osteopenia.

Bone morphology studies by μCT revealed that T1DM affected mostly the trabecular bone compartment, specifically that of the axial skeleton (L5). We found decreases in BV/TV, BMD, and Tb.Th in D‐NOD mice that were associated with increased sclerostin, an inhibitor of bone formation. These observations are in line with findings in adult patients with T1DM (128 men and premenopausal women mean age, 43.4 ± 8.8 years) with a long duration of disease (22.4 ± 9.5 years) that exhibited higher levels of serum sclerostin, irrespective of sex.^(^ ^20^ ^)^ The elevated sclerostin levels could partially explain the blunted bone formation seen in those subjects with T1DM. Another osteocyte‐secreted factor is FGF23 that regulates phosphate and calcium metabolism. According to previous publications, insulin inhibits FGF23 expression via the PI3K/PKB/Akt/FOXO1 pathway,^(39^ ^)^ and insulin deficiency causes a surge in serum concentrations of FGF23.^(^ ^39^ ^)^ Consistent with these observations, we found elevations in osteocyte expression levels of FGF23 in D‐NOD mice that were reduced with insulin. In long bones (femur), bone formation indices in endocortical as well as cancellous bone compartments were significantly reduced in D‐NOD mice by histomorphometry. These were accompanied by reduced gene expression of the osteoprogenitor markers, *osterix* and *twist*, reduced expression of osteocalcin, a marker of osteoblastic activity, and DMP‐1, a marker of early differentiated osteocytes, all together suggesting an impaired osteogenesis. Our data are in accordance with previously reported altered blood flow to bone or the bone marrow of subjects with T1DM,^(^ ^43^ ^)^ which impaired the differentiation niche of osteoprogenitors and consequently blunted the remodeling process. Further support for reduced bone formation was provided by transcriptomics studies that showed marked reduction in the expression of collagen1. We note that mice were followed shortly after development of DM (4 weeks), and the majority of the detected changes in cellular behavior (histomorphometry), cell metabolism (histology), or gene expression (qPCR and RNAseq) were not reflected significantly in bone microarchitecture. Finally, we did not find increases in osteoclast number on bone surfaces of D‐NOD mice. Although we did not study osteoclast activity, it is possible that osteoclasts on bone surfaces of D‐NOD mice were inactive. This is supported by previous reports showing that bone marrow macrophages (the mononuclear progenitors of osteoclasts) in NOD mice have abnormal differentiation caused by an impaired ability to upregulate the receptor for colony‐stimulating factor‐1 (CSF‐1), which is essential for osteoclastogenesis.^(^ ^44^ ^)^ Furthermore, findings in humans also show that T1DM associates mainly with reduced bone formation and not bone resorption.^(^ ^4^, ^20^, ^45^, ^46^ ^)^

Patients with T1DM show relatively modest decreases in BMD (average of −0.5 to −1 SD) as compared with normal individuals.^(^ ^47^ ^)^ These changes cannot fully account for the markedly increased fracture risk in this population. It is commonly hypothesized that alterations in bone quality play major roles in elevated fracture risk in patients with T1DM.^(^ ^48^, ^49^ ^)^ Surprisingly, D‐NOD mice showed reduced mineral:matrix ratio by RS when compared with NOR as early as 4 weeks after detection of DM. Importantly, reduced mineral:matrix ratio occurred in face of blunted bone remodeling within the cortical bone matrix, indicating that it is not an impaired tissue mineralization per se but rather loss of mineral from the bone tissue. Two major possibilities can lead to such a scenario: (i) osteocytes, the major bone cell population that is buried in the bone matrix, undergo osteocytic osteolysis (osteocyte‐driven remodeling of the perilacunar and canalicular matrix,^(^ ^50^ ^)^), such as that which happens during lactation, leading to mineral loss; or (ii) D‐NOD osteocytes are exposed to systemic metabolic alterations that lead to microenvironmental changes favoring mineral loss. The latter possibility is supported by the fact that osteocytes are connected to each other via the lacunar canalicular network, which is interconnected to the vascular canal network and together form conduits throughout the bone matrix. These interconnected networks have a huge fluid surface area that reflects whole‐body metabolism, exposing osteocytes to the metabolic milieu of T1DM. The possibility that hyperglycemia altered osteogenic cell metabolism in the D‐NOD mice is supported by our transcriptome data. Changes in metabolism may lead to accumulation of metabolites (citric acid/lactic acid) favoring mineral loss.

The cells of the osseous system depend heavily on glucose as a source of energy. Osteoblasts metabolize glucose during their differentiation as well as during matrix deposition.^(^ ^51^ ^)^ Insulin‐independent GLUT1 is considered the main glucose transporter expressed in osteogenic cells. We found that osteocytes of D‐NOD mice show elevations in GLUT1 as compared with ND‐NOD mice. Transcriptome data provided additional insights and revealed changes in bone cell metabolism in response to T1DM. One of the key upregulated genes in response to DM, which was also reduced with insulin, was PDK4. The PDK4 enzyme regulates the activity of pyruvate dehydrogenase complex by phosphorylation and inhibits OXPHOS. High levels of PDK4 are seen during starvation and glucose deprivation,^(^ ^52^ ^)^ whereas low levels of PDK4 are detected in response to insulin.^(^ ^53^, ^54^ ^)^ Despite elevated GLUT1 in D‐NOD mice, we found elevated PDK4, indicating a state of cellular starvation, implying that insulin‐dependent glucose uptake does happen in bone cells to some degree. Indeed, GLUT4 immunostaining increased in the In‐NOD group. These data are in accordance with studies using the osteoblast/osteocyte‐specific GLUT4 (insulin‐dependent) KO mice that showed blunted glucose uptake in response to insulin.^(^ ^55^ ^)^ We also found upregulation of genes involved in lipid metabolism in D‐NOD mice. Studies in intact mice have shown that bone cells take up a significant fraction of postprandial lipoprotein.^(^ ^56^ ^)^ Specifically, lipoproteins are required for osteoblast proliferation,^(^ ^57^ ^)^ indicating that osteoblasts or osteocytes also use lipids as an energy source, a process that may also depend on insulin stimulation. However, the molecular insights revealed by RNAseq provide only indirect evidence for the involvement of mitochondrial energy metabolism in the regulation of skeletal integrity and will have to be thoroughly investigated to establish causality.

In summary, we present here morphological, histological, and molecular data from several skeletal sites of a mouse model with naturally occurring T1DM. Perhaps, the most important observation of this study is the reduced mineral:matrix ratio in D‐NOD bones in face of blunted bone formation and unchanged bone resorption. The new molecular findings of metabolic changes and the possible accumulation of metabolites that may favor mineral loss may partially explain why—despite the small changes in BMD in T1DM—there is a significant increase in fracture risk.

## Author Contributions

**Manisha Dixit:** Data curation; formal analysis; methodology. **Zhongbo Liu:** Data curation; methodology. **Sher Bahadur Poudel** Data curation; methodology. **Gozde Yildirim:** Data curation; formal analysis; methodology. **Yanjiao Zhang Zhang:** Data curation. **Shilpa Mehta:** Formal analysis; funding acquisition. **Omer Murik:** Formal analysis. **Geona Altarescu:** Formal analysis; software. **Yoshifumi Kobayashi:** Data curation; methodology. **Emi Shimizu:** Data curation; formal analysis; methodology. **shoshana yakar:** Conceptualization; funding acquisition; investigation; methodology; project administration; supervision; validation; writing‐original draft; writing‐review & editing.

## Conflict of Interest

The authors declare that they have no known competing financial interests or personal relationships that could have appeared to influence the work reported in this article.

## Ethics Approval Statement

Animal protocol was reviewed and approved by the Institutional Animal Care and Use Committees of New York University.

### PEER REVIEW

The peer review history for this article is available at https://publons.com/publon/10.1002/jbm4.10483.

## Supporting information


**Appendix S1**: Supplementary Information

## Data Availability

The data sets generated and analyzed during the current study are available from the corresponding author on reasonable request. Our studies do not include the use of custom code or mathematical algorithms. We have included citations for available data in the references section.
