# Supplementary material for: Skeletal Response to Insulin in the Naturally Occurring Type 1 Diabetes Mellitus Mouse Model
Source: JBMR Plus. 2021 Mar 17;5(5):e10483. doi: 10.1002/jbm4.10483 (PMC8101621; doi:10.1002/jbm4.10483)

Skeletal response to insulin in the naturally occurring type 1 diabetes mellitus mouse model

Manisha Dixit[1], Zhongbo Liu[1], Sher-Bahadur Poudel[1], Gozde Yildirim[1], Yanjiao Zhang Zhang[1], Shilpa Mehta[1], Omer Murik[2], Geona Altarescu[2], Yoshifumi Kobayashi[3], Emi Shimizu[3], Mitchell B Schaffler[4], Shoshana Yakar[1]

[1]David B. Kriser Dental Center, Department of Molecular Pathobiology New York University College of Dentistry New York, NY 10010-4086
[2]Medical Genetics Institute, Shaare Zedek Medical Center, Jerusalem, Israel.
[3]Oral Biology Department, Rutgers School of Dental Medicine, 110 Bergen Street, Newark, NJ, USA.
[4]Department of Biomedical Engineering, City College of New York, 160 Convent Avenue, New York, NY, 10031, USA

Supplements;

Supplemental table 1: Antibodies

Antibody	Company	Catalog no	Dilution used
Cathepsin K antibody	Abcam	ab19027	1:200
pAKT(phospho Ser473) antibody	Genetex	89304-112	1:50
Glut1 antibody	Thermofisher scientific	PA1-46152	1:200
Glut4 antibody	Biogenesis	4670-1704	1:500
PDK4 antibody	Thermofisher scientific	12949-1-AP	1:50
Sclerostin antibody	R & D systems	AF1589	1:12
FGF23 antibody	R & D systems	MAB26291	1:100
Donkey Anti-Goat IgG H&L (HRP)	Abcam	ab97110	1:200
VECTASTAIN® ABC Kit (Rabbit IgG)	Vector labs	PK-4001	NA

Supplemental table 2: qPCR primer sequences

Gene	Forward primer	Reverse primer
Osteocyte markers		
**Phex**	GAAAGGGGACCAACCGAGG	AACTTAGGAGACCTTGACTCACT
**SOST**	AGCCTTCAGGAATGATGCCAC	CTTTGGCGTCATAGGGATGGT
**DMP-1**	TTCGCTGAGGTTTTGACCTT	TTGGGATGCGATTCCTCTAC
**FGF-23**	ATGCTAGGGACCTGCCTTAGA	AGCCAAGCAATGGGGAAGTG
**Pdpn**	ACCGTGCCAGTGTTGTTCTG	AGCACCTGTGGTTGTTATTTTGT
**MEPE**	GTCTGTTGGACTGCTCCTCTT	CACCGTGGGATCAGGATACA
**RANKL**	CAGCATCGCTCTGTTCCTGTA	CTGCGTTTTCATGGAGTCTCA
**OPG**	ACCCAGAAACTGGTCATCAGC	CTGCAATACACACACTCATCACT

Supplement Table 3: mCT analysis of the osseous system of NOR, non-diabetic (*ND*), diabetic (*D*) and insulin treated (*In*) NOD mice. Data presented as mean+/-SD, one-way ANOVA with $p<0.05$ was considered significant. Comparisons that were insignificant are labeled *NS.*

	**NOR**	**Non-diabetic NOD (ND)**	**Diabetic NOD (D)**	**Insulin treated NOD (In)**	**Significance**
**Sample size**	10	26	19	14	Presented $p<0.05$
**Femur mid-diaphysis, cortical bone parameters:**					
**BV/TV, %**	50.039±0.544	57.268±0.409	53.064±0.921	56.237±0.907	NOR vs ND<br>NOR vs D<br>NOR vs In<br>ND vs D
**T.Ar, mm^2**	1.431±0.029	1.416±0.016	1.346±0.014	1.372±0.027	NOR vs D<br>ND vs D
**B.Ar, mm^2**	0.716±0.018	0.811±0.010	0.715±0.016	0.771±0.016	NOR vs ND<br>ND vs D
**M.Ar, mm^2**	0.714±0.014	0.605±0.009	0.631±0.013	0.601±0.019	NOR vs ND<br>NOR vs D<br>NOR vs In
**Cs.Th, mm**	0.183±0.003	0.212±0.002	0.189±0.004	0.205±0.004	NOR vs ND<br>NOR vs In<br>ND vs D<br>D vs In
**J_0, 1/mm^4**	0.251±0.011	0.273±0.006	0.234±0.006	0.252±0.009	NOR vs ND<br>ND vs D
**Length (Le), mm**	15.648±0.127	15.815±0.127	15.316±0.128	15.857±0.065	*NS*
**Robustness (Le/T.Ar)**	0.091±0.002	0.090±0.001	0.088±0.001	0.087±0.002	*NS*
**BMD, g/cc**	1.337±0.004	1.338±0.005	1.348±0.007	1.328±0.006	*NS*
**Femur distal metaphysis, trabecular bone parameters:**					
**BV/TV, %**	6.387±0.402	6.812±0.719	5.818±0.514	5.194±0.883	*NS*
**Tb.Th, mm**	0.057±0.001	0.064±0.001	0.053±0.002	0.060±0.002	NOR vs ND<br>ND vs D<br>D vs In
**Tb.Sp, mm**	0.452±0.016	0.589±0.030	0.549±0.024	0.634±0.031	NOR vs ND<br>NOR vs In
**Tb.N, 1/mm**	1.122±0.061	1.053±0.104	1.097±0.094	0.843±0.123	*NS*
**BMD, g/cc**	0.123±0.005	0.112±0.009	0.112±0.007	0.097±0.013	*NS*
**Lumbar Skeleton (L5):**					
**Sample size**	7	6	6	6	
**BV/TV, %**	20.216±1.030	23.578±1.002	16.447±1.013	15.908±2.014	ND vs D<br>ND vs In
**Tb.Th, mm**	0.059±0.001	0.074±0.002	0.057±0.002	0.061±0.002	NOR vs ND<br>ND vs D<br>ND vs In
**Tb.Sp, mm**	0.259±0.009	0.275±0.009	0.276±0.007	0.315±0.017	NOR vs In
**Tb.N, 1/mm**	3.390±0.140	3.182±0.076	2.881±0.071	2.569±0.207	*NS*
**BMD, g/cc**	0.449±0.017	0.533±0.019	0.375±0.018	0.386±0.032	ND vs D<br>ND vs In
**Alveolar Bone (mandible, between 1st and 2nd Molars)**					
**Sample size**	10	26	21	10	
**BV/TV, %**	52.397±2.387	55.115±3.067	39.636±5.035	62.433±3.101	NOR vs ND<br>NOR vs D<br>NOR vs In<br>ND vs D<br>D vs In
**Tb.Th, mm**	0.095±0.004	0.110±0.005	0.086±0.008	0.119±0.004	NOR vs ND<br>NOR vs In
**Tb.Sp, mm**	0.085±0.005	0.128±0.006	0.145±0.005	0.105±0.011	*NS*
**Tb.N, 1/mm**	5.493±0.149	4.991±0.235	4.482±0.235	5.237±0.108	NOR vs D
**BMD, g/cc**	1.260±0.013	1.419±0.027	1.294±0.043	1.299±0.022	NOR vs ND<br>ND vs D<br>ND vs In

Supplement Figure 1: Gene expression of osteogenic markers at the femur diaphysis, and alveolar bone of the mandible. (A) Expression of osteocyte markers at femur diaphysis. (B) Expression of mature osteoblast and osteocyte markers in alveolar bone. (C) Expression of osteoclast markers in alveolar bone.

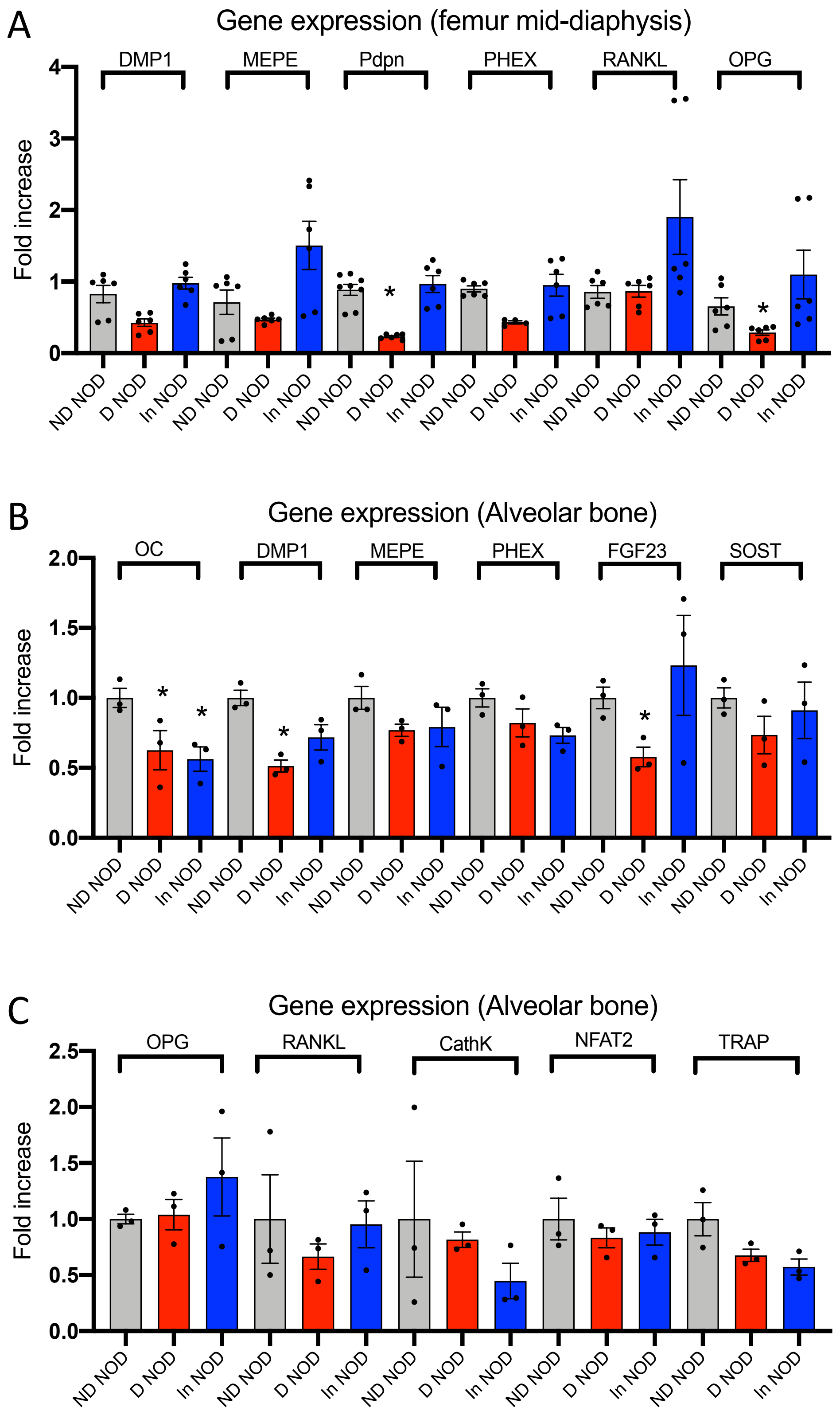

Supplement Figure 2: Diabetic NOD mice show alterations in the expression of GLUT1, PDK4, and p-AKT and GLUT4. GLUT1 (A), PDK4 (B), (C) pAKT, and (D) GLUT4 positive osteocytes in cancellous bone of the L5 vertebra. Representative sections from ND-NOD, D-NOD and In-NOD groups, quantifications are provided in Figure 7.

supplement 2

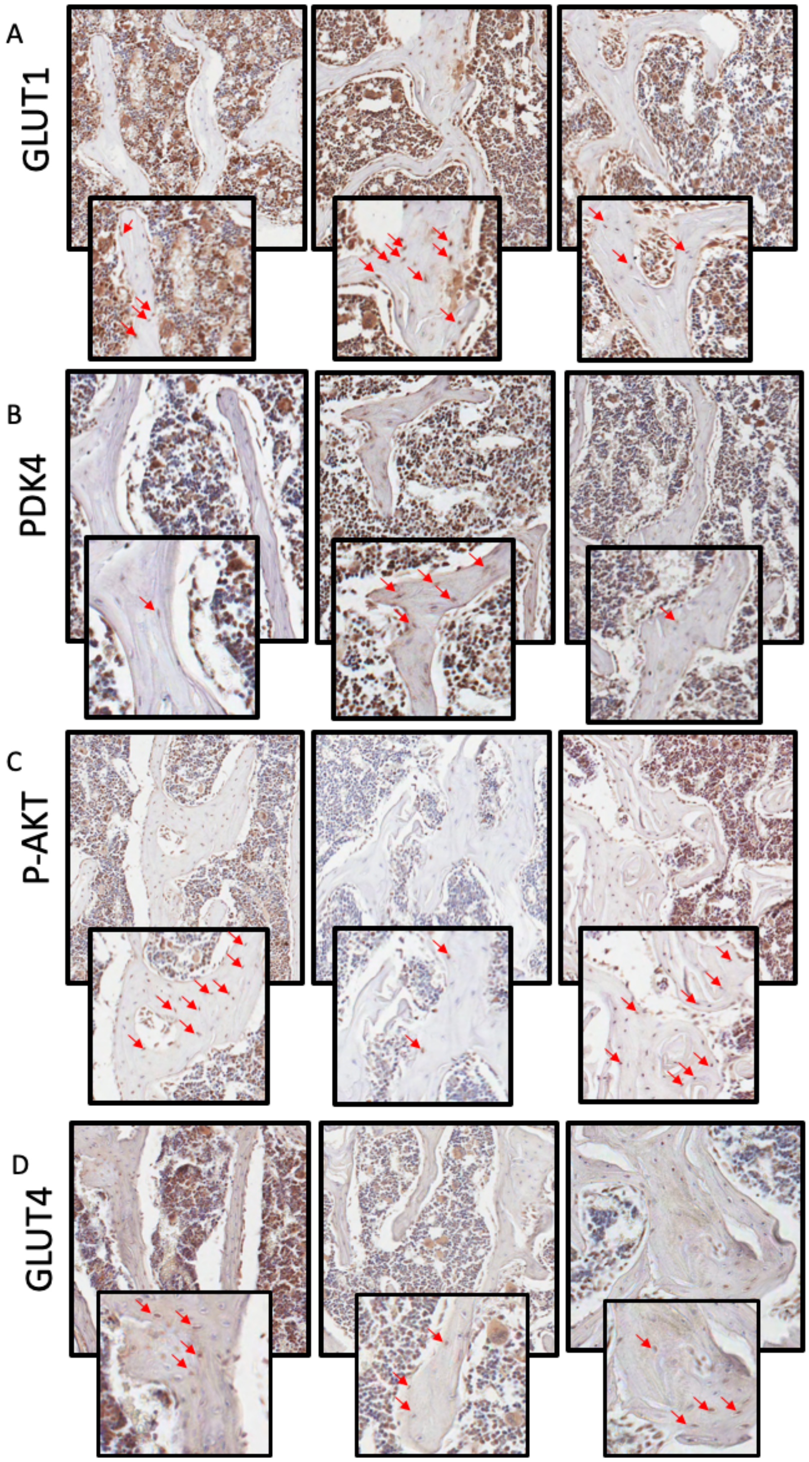

Supplement: Supplementary file 1 — Appendix S1: Supplementary Information [file JBM4-5-e10483-s001.pdf]
